# ﻿*Indosasa fimbriligulata* (Poaceae, Bambusoideae), a new bamboo species from Guangxi, China

**DOI:** 10.3897/phytokeys.265.167921

**Published:** 2025-11-04

**Authors:** Zheng-Yang Niu, Lin Bai, Yi-Hua Tong, Nian-He Xia

**Affiliations:** 1 State Key Laboratory of Plant Diversity and Specialty Crops & Guangdong Provincial Key Laboratory of Digital Botanical Garden, South China Botanical Garden, Chinese Academy of Sciences, Guangzhou, 510650, China South China Botanical Garden, Chinese Academy of Sciences Guangzhou China; 2 South China National Botanical Garden, Chinese Academy of Sciences, Guangzhou, 510650, China South China National Botanical Garden, Chinese Academy of Sciences Guangzhou China

**Keywords:** Arundinarieae, morphology, phylogeny, taxonomy, three-branched bamboos

## Abstract

Based on morphological and molecular phylogenetic evidence, we confirmed that some bamboo collections from Guangxi, China, represent a new species of *Indosasa*. The new species, named *I. fimbriligulata*, is morphologically similar to *I. glabrata* but differs by having internodes that are white strigose when young, and hollow without or with a little clastic pith, culm buds triangular, culm leaf sheaths abaxially scattered with tufted brown setae, culm leaf auricles well developed, and culm leaf ligule apex long fimbriate. A detailed description, as well as two color plates of this new species, is also provided.

## ﻿Introduction

*Indosasa* McClure was established by F. A. McClure (1940), with *I. crassiflora* designated as its generic type. It belongs to the subtribe Arundinariinae of the tribe Arundinarieae (Poaceae, Bambusoideae) (Zhang et al. 2020) and contains 17 species mainly distributed in China (Fujian, Hunan, Guangdong, Guangxi, Guizhou, Yunnan), Vietnam, and Laos (Zhu and Zhao 1996; Zhu and Stapleton 2006; Vorontsova et al. 2016; Niu et al. 2021, 2022, 2025a). It is diagnosed by leptomorph (monopodial) rhizomes, diffuse culms, strongly raised culm supra-nodal ridges, pseudospikelets, six stamens, and three stigmas per floret (McClure 1940; Chao and Chu 1983; Zhu and Zhao 1996; Xue et al. 2003; Zhu and Stapleton 2006; Yi et al. 2008; Xia and Lin 2009; Xia et al. 2016; Shi et al. 2021). However, some recent phylogenomic studies suggested that *Indosasa* sensu lato (s.l.) is non-monophyletic (Guo et al. 2021; Niu 2025). *Indosasa* sensu stricto (s.s.) is defined by the possession of strongly raised and asymmetrically swollen branch supra-nodal ridges, usually 4–9 foliage leaves per ultimate branch, and the traits mentioned above (Niu 2025). The newly defined *Indosasa* s.s. has only eight species, and its distribution is limited to Guangxi, Yunnan, and South Guizhou of China, North Vietnam, and North Laos (Niu 2025).

During two field investigations in Fangchenggang City, Guangxi, we encountered a running bamboo that does not fit the description of any known species. However, some traits, such as strongly raised culm supra-nodal ridges, strongly raised and asymmetrically swollen branch supra-nodal ridges, and the branch complement with three branches per mid-culm node, match well with those diagnostic of *Indosasa* s.s. After comparison with all the previously recorded species of *Indosasa* in national and local floras (Chao and Chu 1983; Zhu and Zhao 1996; Xue et al. 2003; Zhu and Stapleton 2006; Yi et al. 2008; Xia and Lin 2009; Xia et al. 2016; Shi et al. 2021), as well as the monograph of *Indosasa* (Niu 2025), we found that this species is most similar to *I. glabrata* C.D.Chu & C.S.Chao. In order to clarify the taxonomic identity of this bamboo, we conducted phylogenetic and morphological studies. Finally, we confirmed that this bamboo is new to science based on morphological and molecular evidence.

## ﻿Materials and methods

### ﻿Morphological study

Morphological comparisons between this new species and morphologically similar species were conducted based on living plants in the field, specimens, protologues, and descriptions from floras (Chao and Chu 1983; Zhu and Zhao 1996; Zhu and Stapleton 2006; Xia et al. 2016; Niu 2025). Some detailed characters, such as indumentum, were observed with a stereomicroscope (Mshot MZ101, Guangzhou Micro-shot Technology Co., Ltd., Guangzhou, China). Measurements were taken using a ruler or micrometer. Terminology follows McClure (1940), Li et al. (2006), and Beentje (2016).

### ﻿Taxon sampling, DNA extraction, and sequencing

To ascertain the phylogenetic position of this new species, we conducted phylogenetic analyses based on nuclear single-copy orthologous genes (SOGs). According to the most recent phylogenetic framework of three-branched bamboos (Niu et al. 2025b), we selected 22 samples representing 21 bamboo species belonging to eight genera. Specifically, the ingroup from the tribe Arundinarieae contained six species of *Indosasa*, three species of *Acidosasa* B.M.Yang, three species of *Oligostachyum* Z.P.Wang & G.H.Ye, three species of *Pseudosasa* Makino ex Nakai, two species of *Pleioblastus* Nakai, two species of *Sinobambusa* Makino ex Nakai, and one species of *Indocalamus* Nakai. *Bambusa vulgaris* Nees from the tribe Bambuseae was chosen as the outgroup. Sample numbers and their corresponding voucher information are listed in Suppl. material 1: table S1.

For DNA extractions, young leaves were collected in the field and dried with silica gel. Total genomic DNA was extracted from the dried leaves using the Rapid Plus DNA Lib Prep Kit for Illumina (RK2008) (ABclonal, Woburn, USA) and sheared (1 μg DNA per sample) using a Covaris M220 ultrasonicator (Covaris, Woburn, USA). PCR products were purified using the AMPure XP system (Beverly, USA). Library quality was assessed on the Agilent 5400 system (Agilent, USA), and DNA quantification was performed by quantitative polymerase chain reaction (qPCR). The qualified paired-end library was pooled using the NEBNext® Ultra^TM^ DNA Library Prep Kit and sequenced on the NovaSeq 6000 platform with the PE150 strategy. After filtration of adapters and low-quality reads using Fastp software v. 0.23.2 (Chen et al. 2018), we obtained at least 40 Gb of deep genome skimming data.

### ﻿Target nuclear single-copy orthologous genes capture

For nuclear gene recovery, we used the protein-coding sequences of six previously published bamboo genomes – *Dendrocalamus latiflorus* Munro (Zheng et al. 2022), *Phyllostachys edulis* (Carrière) J.Houz. (Zhao et al. 2018), *Bonia amplexicaulis* (L.C.Chia, H.L.Fung & Y.L.Yang) N.H.Xia, *Guadua angustifolia* Kunth, *Olyra latifolia* L., and *Raddia guianensis* (Brongn.) Hitchc. (Guo et al. 2019) – to identify 737 common nuclear single-copy orthologous genes (SOGs) using Orthofinder v. 2.5.4 (Emms and Kelly 2019). We recovered putative SOGs using HybPiper v. 2.0.1 (Johnson et al. 2016). In this pipeline, we employed BWA v. 0.7.17 (Li and Durbin 2009) to map filtered reads to each specified SOG. We then de novo assembled reads and mapped them to each SOG into contigs using SPAdes v. 3.15.0 (Bankevich et al. 2012). We aligned the assembled contigs to the reference SOG dataset and used the Python script ‘retrieve_sequences.py’ to recover 737 putative orthologs for each sample. However, because our samples are polyploid (Guo et al. 2019), some so-called SOGs may have multiple copies. We therefore used the Python script ‘paralog_retriever.py’ to detect and remove potential paralogs. After this step, we retained 479 SOGs.

### ﻿Alignment and phylogenetic inference

We aligned the 479 SOGs using MAFFT v. 7.505 (Katoh and Standley 2013) in Geneious v. 9.1.4 (Kearse et al. 2012). We trimmed each single-gene matrix using trimAl v. 1.4 (Salvador et al. 2009) with default settings. We then removed nuclear genes with lengths shorter than 300 bp or with >50% missing data. The final nuclear dataset used for phylogenetic analyses included 465 SOGs. All SOG alignments can be found in Suppl. material 1.

We used a multi-species coalescent-based method for nuclear gene-based phylogenetic inference. We first inferred individual ML trees using RAxML v. 8.2.12 (Stamatakis 2014) for each nuclear gene and estimated branch support using bootstrapping analysis (Felsenstein 1985) with 500 replicates, all under the GTRGAMMAI model. To improve coalescent analysis accuracy (Zhang et al. 2018; Cai et al. 2021), we collapsed branches with support values below 30% using Newick Utilities v. 1.6.0 (Junier and Zdobnov 2010). We used the resulting collapsed individual gene trees and corresponding bootstrapped trees to infer the species tree based on the multi-locus bootstrapping (MLB) method in ASTRAL-III (Seo 2008; Zhang et al. 2018). We then created 500 coalescent bootstrap replicates, each consisting of 465 gene trees (one tree per gene, randomly sampled from bootstrapping trees without replacement) for quartet analysis. The final MLB species tree was inferred from the 500 resulting “species” trees. MLB branch bootstrap values (BS) were calculated based on the frequency with which branches appeared across the 500 resulting “species” trees using the greedy consensus method (Soto Gomez et al. 2019). We considered BS >70% as strong support.

## ﻿Results

**Figure 1.**
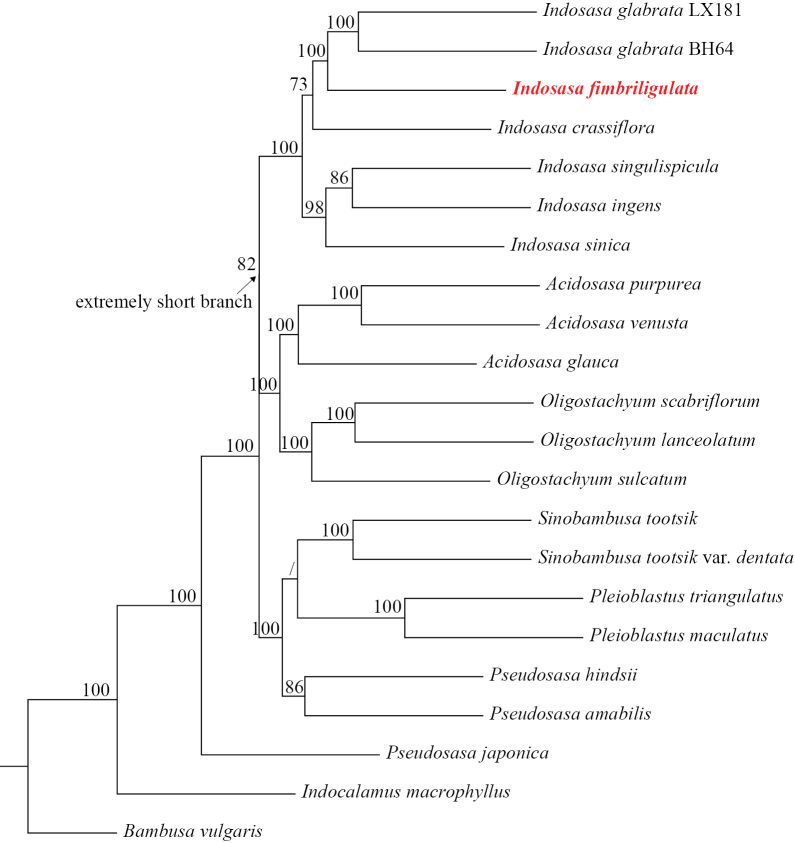
The multi-locus bootstrapping ASTRAL species tree shows the phylogenetic position of *Indosasa fimbriligulata*, which is reconciled by coalescence of 465 single-copy orthologous nuclear gene trees after collapsing branches with support values <30%. BS support values below 70% are represented by a slash.

Our nuclear SOG-based MLB species tree strongly supported that the new species is a member of *Indosasa* s.s. and is closely related to *I. glabrata* (BS = 100) (Fig. 1).

**Figure 2.**
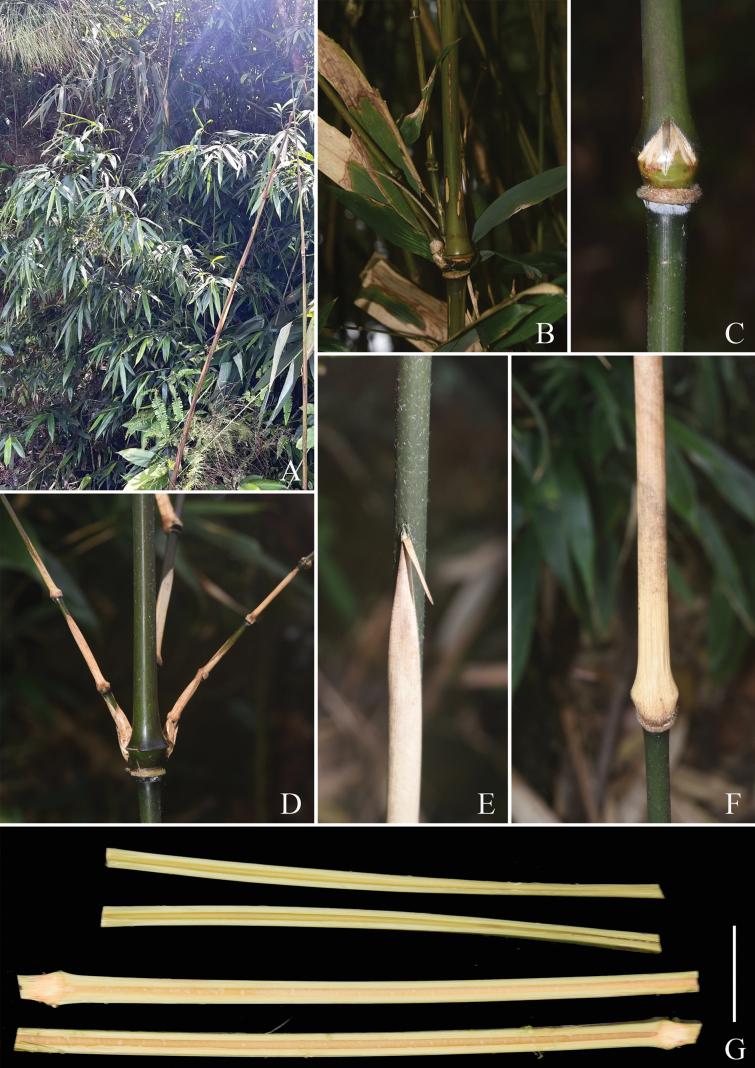
*Indosasa fimbriligulata* N.H.Xia, Z.Y.Niu & Y.H.Tong. A. Habit; B. Old culm; C. Culm bud; D. Branch complement with three branches at culm node; E. Portion of culm leaf showing leaf blade; F. Portion of culm leaf showing sheath base; G. Culm pith. Scale bars: 5 cm (G).

**Figure 3.**
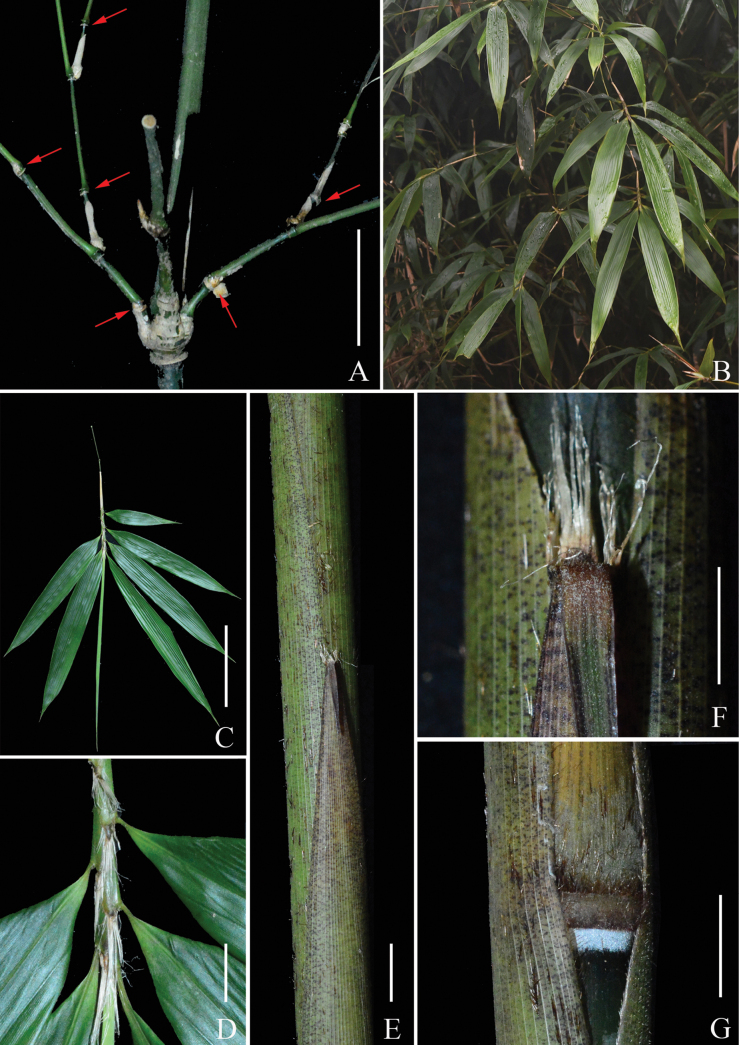
*Indosasa fimbriligulata* N.H.Xia, Z.Y.Niu & Y.H.Tong. A. Branch complement, showing strongly raised and asymmetrically swollen branch supra-nodal ridges; B. Foliage leafy branchlets; C. Ultimate foliage leafy branchlet; D. Portion of foliage leafy branchlet showing auricles and oral setae; E. Portion of culm leaf, showing culm leaf blade; F. Culm leaf ligule with long fimbriae; G. Culm leaf sheath base. Scale bars: 5 cm (A); 10 cm (C); 5 mm (F); 1 cm (D, E, G).

**Figure 4.**
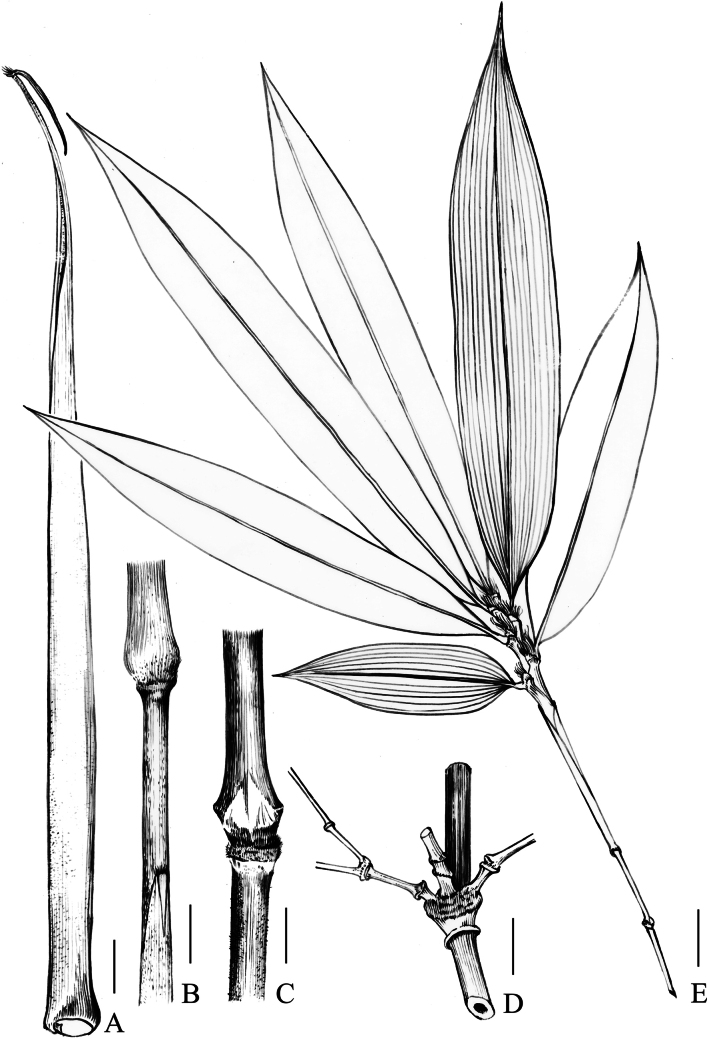
*Indosasa fimbriligulata* N.H.Xia, Z.Y.Niu & Y.H.Tong. A. Culm leaf sheath; B. Portion of culm, showing leaf blade, node, and internode; C. Culm node, showing culm bud and sheath scar; D. Branch complement with three branches at culm node, showing strongly raised and asymmetrically swollen branch supra-nodal ridges; E. Ultimate foliage leafy branchlet. Scale bars: 2 cm (A–E).

Morphological comparison revealed that the new species is morphologically similar to *I. glabrata* in having small culms that are 2–4 m high and 0.5–2 cm in diam., narrow and long triangular papery culm leaf sheaths, and linear-lanceolate culm leaf blades, whereas the other *Indosasa* species are usually characterized by relatively large culms, leathery culm leaf sheaths, and broad lanceolate to ovate culm leaf blades. The new species can be easily distinguished from *I. glabrata* by having triangular (vs. oblate) culm buds, white strigose (vs. glabrous) young culm internodes, without pith or with slightly clastic pith (vs. lamellate pith), abaxial surface of culm leaf sheath with scattered clumps of brown setae and base of culm leaf sheath with dense brown setae (vs. glabrous culm leaf sheath), well-developed (vs. undeveloped) culm leaf auricles and oral setae, and culm leaf ligule with long fimbriae (vs. glabrous) (Table 1; Figs 2–4).

**Table 1.** Morphological comparison of *Indosasa fimbriligulata* and *I. glabrata*.

Characters	I. fimbriligulata	I. glabrata
Young culm		
Primary buds	Triangular	Oblate
Surface of internodes	White strigose	Glabrous
Cavity pith	Nearly absent	Lamellate
Culm leaf		
Abaxial surface of sheaths	Scattered with tufted brown setae	Glabrous
Base of abaxial surface of sheaths	Densely brown setose	Glabrous
Auricles	Small, ca. 0.5 × 1 mm	Undeveloped
Oral setae	Developed, 2–10 mm long	Undeveloped
Apex of ligules	Fimbriate	Glabrous
Blade shape	Linear to linear-lanceolate	Narrow lanceolate to lanceolate

## ﻿Discussion

Although recent phylogenetic studies based on plastid and nuclear sequences confirmed that *Indosasa* s.l. is non-monophyletic (Ma et al. 2017; Guo et al. 2021; Niu 2025), this new species is undoubtedly a member of *Indosasa* s.s. based on both morphological and molecular evidence (Figs 1–4; Table 1). Not only do its morphological characters match well with those of *Indosasa* s.s., but the phylogenetic analysis also showed that it and *I. glabrata* form a monophyletic clade with the type species of *Indosasa*, viz., *I. crassiflora*. Further comparison with morphologically similar species confirmed that it is a distinct new species, which is described and illustrated below.

### ﻿Taxonomic treatment

#### ﻿ Indosasa fimbriligulata

Taxon classificationPlantaePoalesPoaceae

N.H.Xia, Z.Y.Niu & Y.H.Tong sp. nov.

A916FD57-7909-5BF3-840A-5BF3A5D03758

urn:lsid:ipni.org:names:77371342-1

Figs 2, 3, 4

##### Type.

China • Guangxi Zhuang Autonomous Region: Fangchenggang City, Naliang Town, Gaolin Village; 21°37'48"N, 107°40'57"E; alt. 391 m; 17 April 2025; *L. Bai & H. Y. Li BLSC-25041501* (holotype: IBSC!).

##### Diagnosis.

*Indosasa fimbriligulata* resembles *I. glabrata* but differs from the latter in having white strigose internodes when young, hollow internodes without pith or with a little clastic pith, abaxial surface of culm leaf sheaths scattered with tufted brown setae, well-developed culm leaf auricles, and culm leaf ligule apex with long fimbriae.

##### Description.

Rhizomes leptomorph. Culms diffuse, erect, sometimes flexuose, 2–4 m tall and 0.5–1.5 cm in diameter; internodes flattened on branching side, with two longitudinal ridges and three grooves above branching points along the branching side, 30–50 cm long, initially green to dark green, yellow-green when aged, white strigose when young, glabrescent when old, white powdery only at infranodal regions; walls 3–4 mm thick, cavity hollow without pith or with slightly clastic pith; supra-nodal ridges strongly raised, sheath scars prominent, with a corky ring and densely brown setose when young, glabrescent when old, with persistent remains of sheath base. Branch supra-nodal ridges strongly raised and asymmetrically swollen. Primary buds solitary, triangular, yellowish green, not sunken into culm, margin densely ciliate. Branch complement with three branches at each culm node. Culm leaf sheaths initially green with small purple dots, more so on the marginal part, turning to straw when old, tardily deciduous, narrow and long triangular, papery, abaxially scattered with tufted brown setae, densely brown setose at base, margins densely ciliate; auricles small, ovate to triangular, ca. 1 × 0.5 mm; oral setae well-developed, many, straight or slightly tortuous, 2–10 mm long; ligules arcuate, ca. 2 mm tall, abaxially puberulent, apex long fimbriate, fimbriae many, straight or slightly tortuous, 3–7 mm long; blades reflexed, linear to linear-lanceolate, 0.8–3 × 0.1–0.3 cm, 3/50–6/50 as long as sheath, apex acuminate, base narrowed, adaxially sparsely brown setae, abaxially glabrous. Foliage leaves 4–7 per ultimate branchlet; sheaths 3.5–6.5 cm long, glabrous, longitudinal ribs conspicuous when dry; auricles well-developed, ovate to triangular, 3–5 × 1–3 mm; oral setae many, straight or slightly tortuous, 3–20 mm long; ligules short, truncate, 0.5–1 mm tall, abaxially pubescent; blades lanceolate, papery, 9.5–24 × 1.5–3.8 cm, apex acuminate, base cuneate to widely cuneate, glabrous on both sides, one margin serrulate, other margin entire, secondary veins 5–8 pairs, transverse veinlets conspicuous. Inflorescence unknown.

##### Phenology.

New shoots from April to June.

##### Distribution and habitat.

 This new species has been found only in its type locality so far. It usually occurs on mountain slopes near roadsides and streams.

##### Etymology.

The specific epithet refers to the fimbriate culm leaf ligule of this species, which is rarely seen in other *Indosasa* species. Its Chinese name is given as 流苏大节竹 [liú sū dà jié zhú].

##### Additional specimens examined.

**China • Guangxi**: Fangchenggang City, Naliang Town, Gaolin Village; 21°37'34"N, 107°41'8''E; alt. 443 m; 15 June 2025; *Z. Y. Niu*, *NZY297* (IBSC).

##### Local usage.

Old culms are used for fences.

## Supplementary Material

XML Treatment for Indosasa fimbriligulata

## ﻿Additional information

### Use of AI

No use of AI was reported.

### Author ORCIDs

Zheng-Yang Niu https://orcid.org/0000-0003-0281-1504

Yi-Hua Tong https://orcid.org/0000-0002-5034-005X

Nian-He Xia https://orcid.org/0000-0001-9852-7393

### Data availability

All of the data that support the findings of this study are available in the main text or Supplementary Information.

## Supplementary materials

Supplementary material 1 465 single-copy orthologous gene alignments used for the reconstruction of MLB ASTRAL species tree and one supplementary table Data type zip Explanation note Here are 465 single-copy orthologous gene alignments used for the reconstruction of multi-locus bootstrapping ASTRAL species tree, and their corresponding specimen voucher information used in our study. https://binary.pensoft.net/file/1457360This dataset is made available under the Open Database License (http://opendatacommons.org/licenses/odbl/1.0/). The Open Database License (ODbL) is a license agreement intended to allow users to freely share, modify, and use this Dataset while maintaining this same freedom for others, provided that the original source and author(s) are credited.Author: Zheng-Yang Niu
